# Observed switches and derived profitability indicators for peaking power plants: Northeast U.S. 2001–2009

**DOI:** 10.1016/j.dib.2019.104034

**Published:** 2019-05-24

**Authors:** Stein-Erik Fleten, Erik Haugom, Alois Pichler, Carl J. Ullrich

**Affiliations:** a Norwegian University of Science and Technology, Norway; b Inland Norway University of Applied Sciences, Norway; c James Madison University, USA

## Abstract

The data are related to the research article “Structural estimation of switching costs for peaking power plants,” https://doi.org/10.1016/j.ejor.2019.03.031. Fleten et al., 2019 We display the operating status of peaking power plants as they were reported annually to the United States Energy Information Administration during 2001–2009. Operating status can either be operating, on standby, or retired. Changes in operating status allow us to infer shutdowns, startups, and retirements. We also derive annual profitability indicators.

**Table:** Specifications table

Subject area	*Business Economics*
More specific subject area	*Real Options*
Type of data	*Table in worksheet*
How data was acquired	*Web download*
Data format	*Filtered, processed*
Experimental factors	*We infer status changes (shutdown, startup, and retirement) from annual plant status data.*
Experimental features	*We combine generator data from EIA860 with (i) electricity prices, (ii) fuel prices, (iii) heat rate information, (iv) interest rates, and, (v) nonfuel variable and fixed costs to calculate an annual profitability indicator for each plant.*
Data source location	*Energy Information Administration (EIA); PJM, NEISO, NYISO electricity market areas; Environmental Protection Agency (EPA) Air Markets Program Data (AMPD); EIA Annual Energy Outlook Assumptions*
Data accessibility	1) EIA Form 860 (https://www.eia.gov/electricity/data/eia860/) [2] 2) Electricity prices from ISO websites (https://dataminer2.pjm.com/list) [3], (https://www.iso-ne.com/isoexpress/web/reports/pricing) [4], (https://www.nyiso.com/energy-market-operational-data) [5] 3) fuel prices from EIA (https://www.eia.gov/naturalgas/data.php#prices) [6], (https://www.eia.gov/petroleum/) [7] 4) Heat rates from CEMS database of EPA (https://ampd.epa.gov/ampd/) [8] 5) Non-fuel generation costs from AEO of EIA (https://www.eia.gov/outlooks/aeo/assumptions/) [9]
Related research article	*Fleten, S-E, E. Haugom, A. Pichler, and C. Ullrich, Structural estimation of switching costs for peaking power plants, European Journal of Operational Research, forthcoming 2019.*[1]

**Table:** 

**Value of the data** • Peaking power plants are essential to electric system reliability as they provide reserve generation. Status change information therefore supports analysis of system reliability. • Profitability indicators allow comparisons across plants be they operating or shutdown. • This is data from the U.S., but comparing these with similar data from Europe and elsewhere could give additional insight, particularly if data for other types of generators are found. • The data includes retirements, which could be interesting on its own as the basis an empirical studies. The same applies to mothballing.

## 1 Data

The following data items are included in the data table in the accompanying spreadsheet.

• year
• ngen (plant identifier)
• status (OP – operating, SB – shutdown, RE - retired)
• outgoing status (OP – operating, SB – shutdown, RE - retired)
• status change (shutdown, startup, retirement)
• heat rate (MMBtu/MWh)
• summer capacity (MW)
• age (years)
• fuel type (NG = natural gas, DFO = oil)
• state
• region (PJM, ISO-NE, NYISO)
• profitability indicator ($/kW/year)

**Fig. 1:**
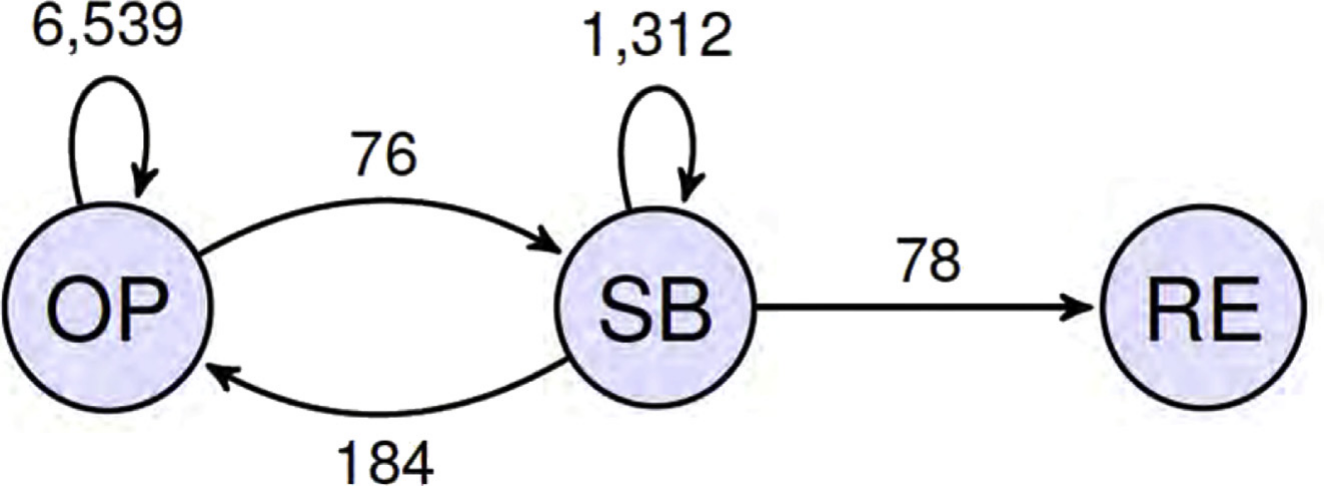
Transitions between the three states operating (OP), standby (SB), and retired (RE). Included is the number of observed transitions. Graph from Fleten et al. (2019) [1].

The sample includes simple cycle combustion turbines (CT) located in Connecticut, Delaware, Illinois, Indiana, Kentucky, Maine, Maryland, Massachusetts, Michigan, New Hampshire, New Jersey, New York, North Carolina, Ohio, Pennsylvania, Rhode Island, Tennessee, Vermont, Virginia, Washington D.C., and West Virginia. The data set contains 8189 plant-year observations on 1121 individual plants. Fig. 1 displays transitions in the “status” variable.

## 2 Experimental design, materials and methods

Preprocessing of the data consists of matching generator names/ID numbers between the various databases. The main data items are (i) status changes, and, (ii) profitability indicators for peaking power plants in the United States. The primary data source is Form 860 collected and disseminated by the Energy Information Administration (hereafter EIA) [2]. These data are supplemented by electricity prices (PJM, NYISO, ISO-NE websites) [3], [4], [5], fuel prices (EIA) [6], [7], heat rates (US EPA CEMS data) [8], and nonfuel costs (EIA AEO supplemental data) [9].

The possible values for the status variable in EIA860 are.

• OP – operating,
• SB – shutdown (mothballed, in cold standby), and,
• RE – retired.

We define a **shutdown** to occur when a plant moves from state OP to state SB. We define a **startup** to occur when a plant moves from state SB to state OP. We define a **retirement** to occur when a plant moves from state SB to state RE. Other possible (non)transitions include OP to OP and SB to SB.

The spark spread on day *n* is equal to the value of electricity generated minus the cost to generate the electricity.

(1) S_n_ = P^e^_n_ – H*P^f^_n_ – V

Where,

*S*_*n*_ = spark spread on day *n* in units of *$/MWh*,

*P*^*e*^_*n*_ = day *n* electricity price in units of *$/MWh*,

*H* = heat rate in units of *MMBtu/MWh*,

*P*^*f*^_*n*_ = day *n* fuel price in units of *$/MMBtu*, and

*V* = nonfuel variable O&M (operations and maintenance) costs.

For each generator we calculate daily spark spread, then sum the positive spreads over the days of the year (*T*_*t*_) to obtain an annual profitability indicator for year *t, X*_*t*_, in units of $/kW/year.

(2) $$X_{t}=\sum_{n=1}^{T_{t}}\max\left(S_{n},0\right)*\left(\frac{16}{1000}\right)$$

The profitability indicator is the theoretical annual profit per unit of generating capacity. The max function captures the optionality of the plant. We assume that the plant would not run on days for which the spread is negative. 16 is the number of hours is the peak period for one day and 1000 converts MW to kW.
