# Supplementary material for: Observed switches and derived profitability indicators for peaking power plants: Northeast U.S. 2001–2009
Source: Data Brief. 2019 May 24;25:104034. doi: 10.1016/j.dib.2019.104034 (PMC6554235; doi:10.1016/j.dib.2019.104034)
Supplement: Supplementary file 1 — Multimedia component 1 [file mmc1.docx]

May 7, 2019

Dear Editor:

- All authors have participated in (a) conception and design, or analysis and interpretation of the data; (b) drafting the article or revising it critically for important intellectual content; and (c) approval of the final version.
- This manuscript DIB-D-19-00816 has not been submitted to, nor is under review at, another journal or other publishing venue.
- The authors have no affiliation with any organization with a direct or indirect financial interest in the subject matter discussed in the manuscript

Sincerely,

Stein-Erik Fleten

Erik Haugom

Alois Pichler

Carl J. Ullrich
